# The complete chloroplast genome sequence of *Salvia miltiorrhiza*, a medicinal plant for preventing and treating vascular dementia

**DOI:** 10.1080/23802359.2020.1778574

**Published:** 2020-06-16

**Authors:** Jiulue Hu, Miao Zhao, Zijun Hou, Jian Shang

**Affiliations:** a Zhang Zhongjing College of Chinese Medicine, Nanyang Institute of Technology, Nanyang, China; b Henan Key Laboratory of Zhang Zhongjing Formulae and Herbs for Immunoregulation, Nanyang, China; c College of Basic Medicine, Henan University of Traditional Chinese Medicine, Zhengzhou, China

**Keywords:** *S. miltiorrhiza*, chloroplast genome, phylogenetic analysis, genetic information

## Abstract

*S. miltiorrhiza* is a perennial herb of the genus *Salvia* (Lamiaceae), which is an important medicinal plant for preventing and treating vascular dementia. The complete chloroplast genome sequence of *Salvia miltiorrhiza* was characterized from Illumina pair-end sequencing. The chloroplast genome of *S. miltiorrhiza* was 152,680 bp in length, containing a large single-copy region (LSC) of 84,104 bp, a small single-copy region (SSC) of 17,638 bp, and two inverted repeat (IR) regions of 25,469 bp. The overall GC content is 38.70%, while the correponding values of the LSC, SSC, and IR regions are 36.2%, 31.9%, and 43.2%, respectively. The genome contains 131 complete genes, including 86 protein-coding genes (62 protein-coding gene species), 37 tRNA genes (29 tRNA species) and 8 rRNA genes (4 rRNA species). The Neighbour-joining phylogenetic analysis showed that *S. miltiorrhiza* and *Salvia przewalskii* clustered together as sisters to other *Salvia* species.

## Introduction

**Figure 1.**
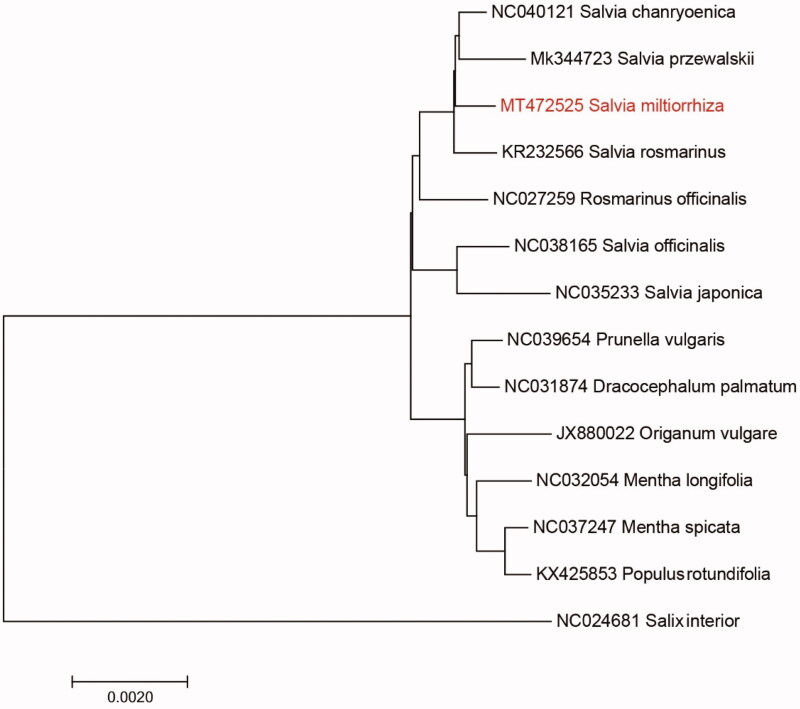
Neighbour-joining (NJ) analysis of *S. miltiorrhiza* and other related species based on the complete chloroplast genome sequence.

*Salvia miltiorrhiza* is a perennial herb of the genus *Salvia* (Lamiaceae), which is an important medicinal plant for preventing and treating vascular dementia and widely distributed in China, which has persisted largely in an undomesticated state that is highly resistant to different environmental stresses. Vascular dementia refers to a disease caused by cerebrovascular disorders, with dementia as the main clinical phase. Modern pharmacological studies have shown that *S. miltiorrhiza* can significantly increase the number of capillary networks and accelerate blood flow, thereby restoring the function of microcirculation. At the same time, it can also reduce plasma lactic acid content and improve metabolic disorders caused by cell hypoxia. It plays an important role in preventing and treating vascular dementia. Studies have shown that *S. miltiorrhiza* can reduce the content of lipid peroxide and erythrocyte sorbitol, increase the level of superoxide dismutase, reduce the oxidative stress reaction, and has an antioxidant effect. Studies have also found that *S. miltiorrhiza* can improve the role of vascular dementia. The mechanism may be: the antioxidant effect of drugs, by restoring the structure and function of nerve cells, inhibiting lipid peroxidation, improving blood rheology, and protecting against ischemia. Damaged neurons improve the pathological response of vascular dementia. *S. miltiorrhiza* has high ecological and economic value with high levels of intraspecific genetic diversity. *S. miltiorrhiza* has wide geographic distribution, high intraspecific polymorphism, adaptability to different environments, combined with a relatively small genome size. Consequently, *S. miltiorrhiza* represents an excellent model for understanding how different evolutionary forces have sculpted the variation patterns in the genome during the process of population differentiation and ecological speciation (Neale and Antoine 2011). Moreover, we can develop conservation strategies easily when we understand the genetic information of *S. miltiorrhiza*. In the present research, we constructed the whole chloroplast genome of *S. miltiorrhiza* and understood many genome varition information about the species, which will provide beneficial help for population genetics studies of *S. miltiorrhiza* (Figure 1).

The fresh leaves of *S. miltiorrhiza* were collected from Chengdu (102°54′E; 30°05′N). Fresh leaves were silica-dried and taken to the laboratory until DNA extraction. The voucher specimen (SDH001) was laid in the Herbarium of Nanyang Institute of Technology and the extracted DNA was stored in the −80 °C refrigerator of the Key Laboratory of School of Biological and Chemical Engineering. We extracted total genomic DNA from 25 mg silica-gel-dried leaf using a modified CTAB method (Doyle 1987). The whole-genome sequencing was then conducted by Biodata Biotechnologies Inc. (Hefei, China) with Illumina Hiseq platform. The Illumina HiSeq 2000 platform (Illumina, San Diego, CA) was used to perform the genome sequence. We used the software MITObim 1.8 (Hahn et al. 2013) and metaSPAdes (Nurk et al. 2017) to assemble chloroplast genomes. We used *Salvia przewalskii* (GenBank: MK344723) as a reference genome. We annotated the chloroplast genome with the software DOGMA (Wyman et al. 2004), and then corrected the results using Geneious 8.0.2 (Campos et al. 2016) and Sequin 15.50 (http://www.ncbi.nlm.nih.gov/Sequin/).

The complete chloroplast genome of *S. miltiorrhiza* (GenBank accession number MT472525) was characterized from Illumina pair-end sequencing. The complete chloroplast genome sequence of *Salvia miltiorrhiza* was characterized from Illumina pair-end sequencing. The chloroplast genome of *S. miltiorrhiza* was 152,680 bp in length, containing a large single-copy region (LSC) of 84,104 bp, a small single-copy region (SSC) of 17,638 bp, and two inverted repeat (IR) regions of 25,469 bp. The overall GC content is 38.70%, while the correponding values of the LSC, SSC, and IR regions are 36.2%, 31.9%, and 43.2%, respectively. The genome contains 131 complete genes, including 86 protein-coding genes (62 protein-coding gene species), 37 tRNA genes (29 tRNA species) and 8 rRNA genes (4 rRNA species).

We used the complete chloroplast genomes sequence of *S. miltiorrhiza* and 13 other related species to construct phylogenetic tree. The 14 chloroplast genome sequences were aligned with MAFFT (Katoh and Standley 2013), and then the Neighbour-joining tree was constructed by MEGA 7.0 (Kumar et al. 2016). The Neighbour-joining phylogenetic analysis showed that *S. miltiorrhiza* and *Salvia przewalskii* clustered together as sisters to other *Salvia* species.

## Data Availability

The data that support the findings of this study are openly available in GenBank at https://www.ncbi.nlm.nih.gov, reference number MT472525.
